# Supplementary material for: A Transparent Electrode Based on Solution-Processed ZnO for Organic Optoelectronic Devices
Source: Nat Commun. 2022 Jul 28;13:4387. doi: 10.1038/s41467-022-32010-y (PMC9334612; doi:10.1038/s41467-022-32010-y)
Supplement: Supplementary file 1 — Supplementary Information [file 41467_2022_32010_MOESM1_ESM.pdf]

Supplementary Information

A Transparent Electrode Based on Solution-Processed ZnO for Organic Optoelectronic Devices

Chen et al.

Supplementary Note 1

The V_{oc} of an organic solar cell is primarily determined by the energy of the bandgap of the active layer (E_g) [*Phys. Rev. B* 81, 125204 (2010)]. In this work, we determine E_g using the normalized reduced absorption and emission spectra of the active layer [*Sustain. Energy Fuels* 2, 538-544 (2018)], and we find that the E_g values are the same for the PBDB-T:ITIC active layers on ZnO and ITO (Supplementary Figure 7a). Then, the difference in V_{oc} must be a result of different voltage losses, according to the equation [*Phys. Rev. B* 81, 125204 (2010)]:

$$V_{oc} = V_{oc,rad} - \Delta V_{nr} = \frac{E_g}{q} - \Delta V_r - \Delta V_{nr} \qquad (1)$$

where q is the elementary charge, $V_{oc,rad}$ (= $\frac{E_g}{q} - \Delta V_r$) is the radiative recombination limit for V_{oc}, and ΔV_r and ΔV_{nr} are the radiative and non-radiative voltage losses, respectively. $V_{oc,rad}$ and ΔV_r in organic solar cells are mostly related to the energetics and the decay dynamics of the charge-transfer (CT) state. Therefore, the energetic properties of the CT state are characterized using sensitive EQE and electroluminescence (EL) spectroscopies. From the sensitive EQE and EL spectra, shown in Supplementary Figure 7b, we find that the low energy absorption and emission, related to the CT state electronic properties, are similar for the solar cells based on ZnO and ITO.

Using the analytical equation derived in the Marcus theory [*Phys. Rev. B* 81, 125204 (2010)]:

$$EQE(E) = \frac{f}{E\sqrt{4\pi kT}} \exp\left(\frac{-(E_{CT}+\lambda-E)^2}{4\lambda kT}\right) \qquad (2)$$

where k is the Boltzmann constant, T is temperature, λ is the reorganization energy of CT states, E is photon energy, and f is a prefactor related to the absorption oscillator strength of CT states, we find that the energy of CT states (E_{CT}) is 1.47 eV and λ is 0.39 eV for both the solar cells based on ZnO and ITO. These results suggest that the CT state energetic properties are not affected by the replacement of ITO with ZnO, as expected. Therefore, the $V_{oc,rad}$, and thus, ΔV_r should be similar for the solar cells based on ZnO and ITO.

To determine $V_{oc,rad}$ and ΔV_r for the solar cells based on ZnO and ITO, we first calculate $J_{0,rad}$, the radiative limit for the saturation current of the solar cell, using the equation [*Phys. Rev. B* 81, 125204 (2010)]:

$$J_{0,rad} = q \int EQE(E) BB(E)\, dE \qquad (3)$$

where *BB(E)* is the blackbody photon flux at 300 K. The calculated $J_{0,rad}$ values are listed in **Table 1** of the main article. Then, we calculate $V_{oc,rad}$, using the equation [*Phys. Rev. B* 81, 125204 (2010)]:

$$V_{oc,rad} = \frac{kT}{q} \ln\left(\frac{J_{ph}}{J_{0,rad}}\right) \qquad (4)$$

where J_{ph} is the photocurrent density in the solar cell under open circuit, assumed to be equal to J_{sc}. Now, we calculate ΔV_r, using the equation,

$$\Delta V_r = \frac{E_g}{q} - V_{oc,rad} \qquad (5)$$

As listed in Table 1, we find that the use of the ZnO stack, instead of ITO, indeed does not lead to a change in $V_{oc,rad}$ or ΔV_r.

Finally, we determine ΔV_{nr} for the solar cells based on ZnO and ITO, using equation (1). The results are also listed in Table 1: We find that ΔV_{nr} of the solar cell based on ZnO is much lower than that based on ITO (0.31 V *vs* 0.21 V), which is the main reason for the higher V_{oc} of the ZnO based solar cell. Because ΔV_{nr} is related to the device electroluminescence quantum efficiency (EQE_{EL}) [*Phys. Rev. B* 81, 125204 (2010)],

$$\Delta V_{nr} = -\frac{kT}{q}\ln(EQE_{EL}) \qquad (6)$$

EQE_{EL} of the solar cells based on ZnO and ITO must be different. This is indeed the case according to the EQE_{EL} measurements (Supplementary Figure 7c): We find that EQE_{EL} for the solar cell based on ZnO is 0.1 % (determined at the injection current density close to J_{sc}), much higher than that based on ITO (0.02 %).

In organic solar cells, EQE_{EL} is determined by the ratio between the radiative (k_r) and non-radiative recombination rate (k_{nr}) of the photogenerated charge carriers [*Annu. Rev. Phys. Chem.* 67, 113–133 (2016)],

$$EQE_{EL} = \frac{k_r}{k_r+k_{nr}} \approx \frac{k_r}{k_{nr}} \quad (for\ k_{nr} \gg k_r) \qquad (7)$$

Because ΔV_r (logarithmically dependent on k_r) is found similar for the solar cells based on ZnO and ITO, k_r should also be similar in these solar cells. Then the difference in EQE_{EL} must be due to different k_{nr}, which is determined by the density of the non-radiative decay channels in the active layer.

Supplementary Figures

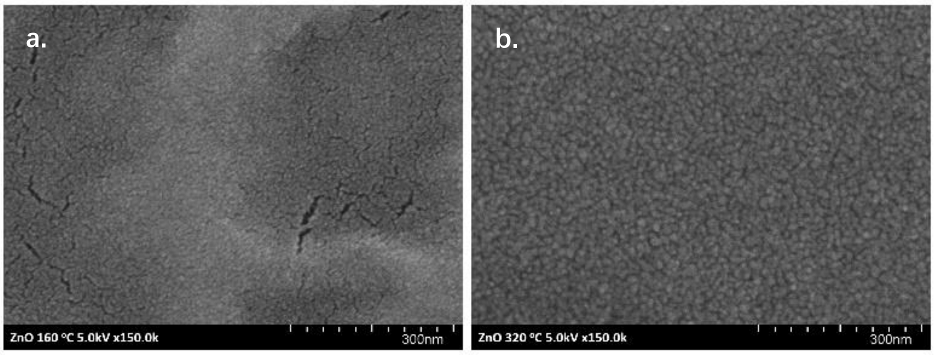

Supplementary Figure 1. SEM images of the sol-gel-grown ZnO films annealed at **a)** 160 ^{o}C and **b)** 320 ^{o}C.

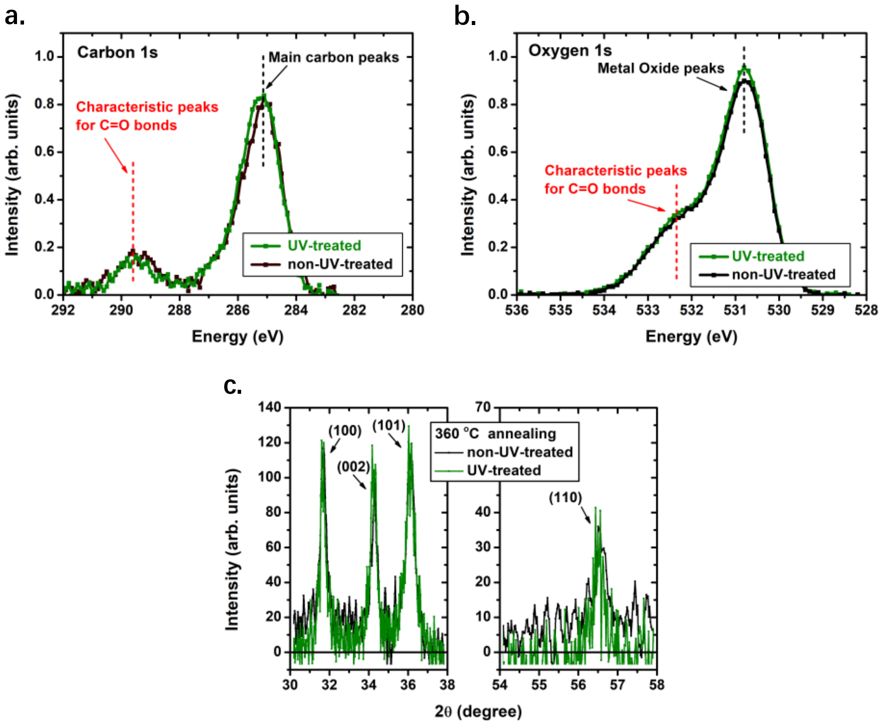

Supplementary Figure 2. a) XPS Carbon 1s peaks and **b)** XPS oxygen 1s peaks for the single-layer ZnO films annealed at 320 ^{o}C, before and after UV treatment (365 nm, 24 W, 600 s). **c)** XRD spectra for the single-layer ZnO films annealed at 360 ^{o}C, before and after UV treatment.

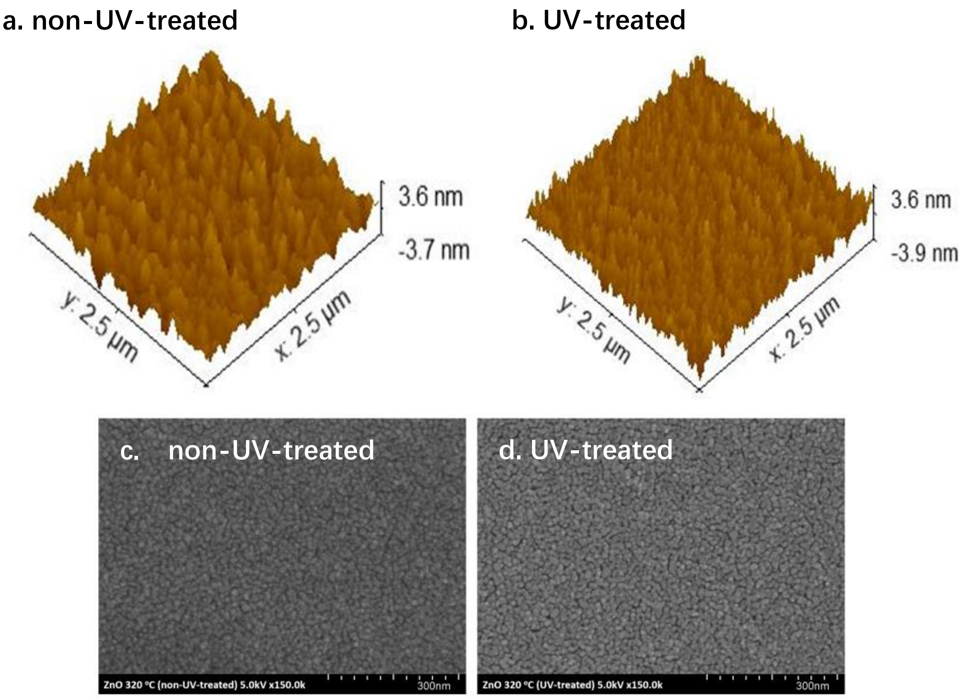

Supplementary Figure 3. AFM images for the single-layer ZnO annealed at 320 °C, **a)** before and **b)** after UV treatment (365 nm, 24 W, 600 s). SEM images for the single-layer ZnO annealed at 320 °C, **c)** before and **d)** after UV treatment.

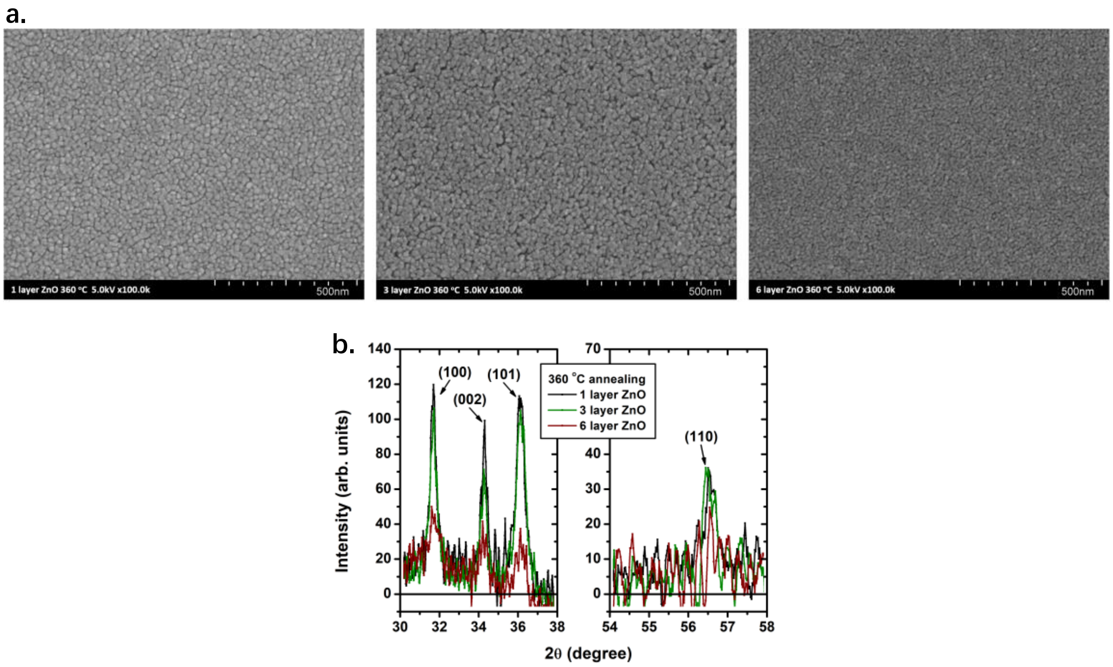

Supplementary Figure 4. a) SEM and **b)** XRD measurements for the ZnO stacks with different numbers of ZnO layers annealed at 360 °C. From the SEM images, we observe that the size of the ZnO nanocrystals reduces with the increasing number of ZnO layer in stack, and from the XRD spectra, we find that the intensity of the diffraction peaks reduces with the increasing number of ZnO layer in the stack. These results suggest that the repeated intensive thermal treatments for growing the high temperature annealed ZnO stack lead to a structural change of the ZnO films in the stack.

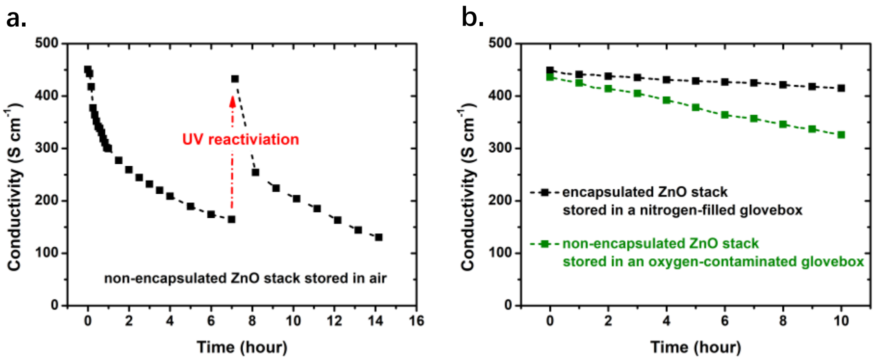

Supplementary Figure 5. a) Conductivity of a non-encapsulated ZnO stack with 6 layers annealed at 320 ºC after UV treatment (365 nm, 24 W, 600 s), stored in air. **b)** Conductivity of ZnO stacks with 6 layers annealed at 320 ºC after UV treatment, stored in a nitrogen-filled glovebox and an oxygen-contaminated glovebox (oxygen concentration: 800 ppm).

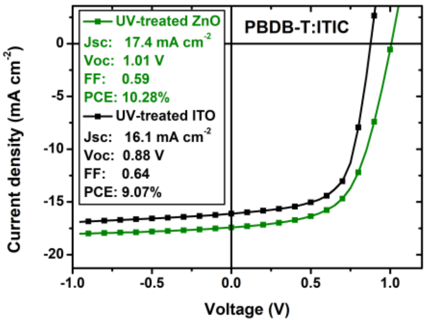

Supplementary Figure 6. *J-V* curves and the performance parameters for the UV-treated solar cells based on ITO, compared to the UV-treated solar cells based on the ZnO stack with 6 layers of ZnO, annealed at 320 ºC, with the active layers based on PBDB-T:ITIC.

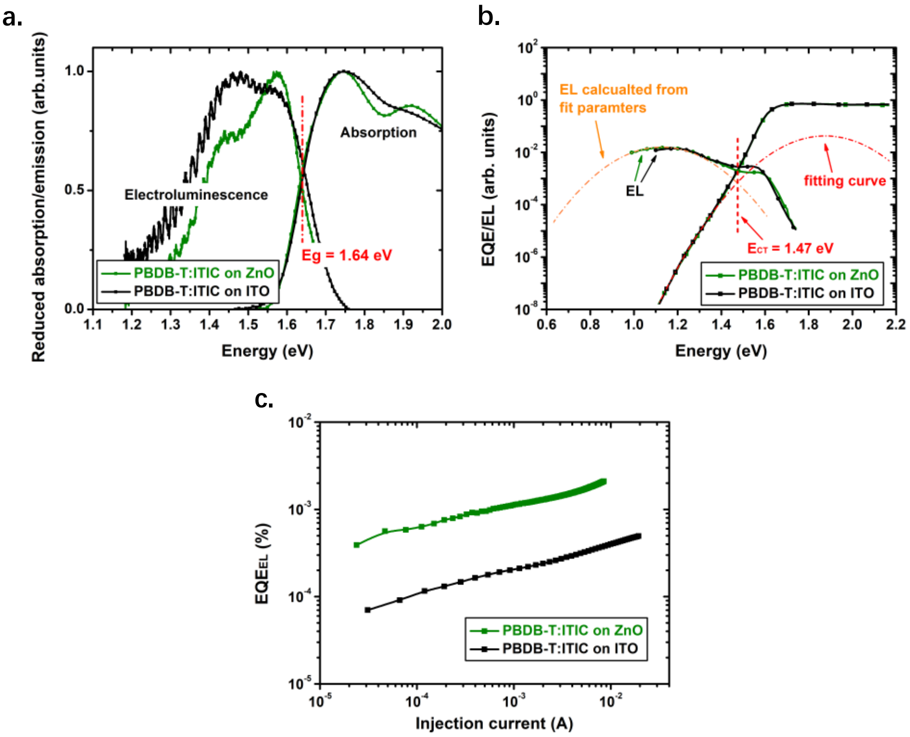

Supplementary Figure 7. Exciton energetics in the PBDB-T:ITIC solar cells based on ZnO and ITO. **a)** Normalized reduced absorption and emission spectra of the active layers of PBDB-T:ITIC in the solar cells based on the ZnO stack and ITO. **b)** Sensitive photovoltaic EQE and EL spectra of the solar cells based on ZnO and ITO. The low energy part of the spectra, corresponding to CT state absorption and emission are evaluated using the equation derived in the framework of Marcus theory, for the determination of E_{CT} and λ. **c)** EQE_{EL} of the solar cells based on ZnO and ITO. The origin for the different V_{oc} of the ZnO and the ITO based solar cells are discussed below.

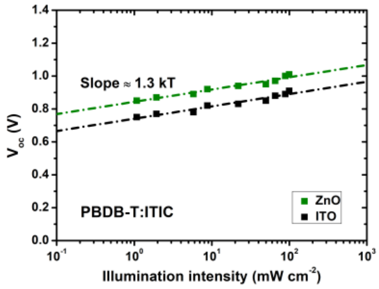

Supplementary Figure 8. V_{oc} of the solar cells based on ZnO and ITO, with the active layer based on PBDB-T:ITIC, measured as a function of illumination intensity. The slope of V_{oc} *vs* intensity is about 1.3 kT for both the ZnO and the ITO based solar cells, suggesting that the trap-assisted recombination losses in these solar cells are weak.

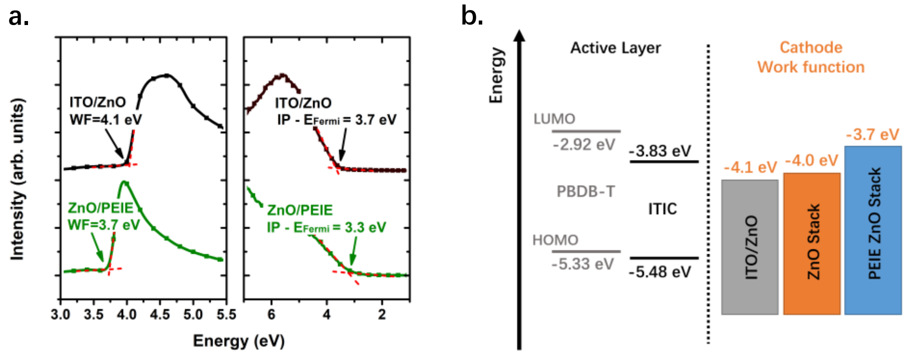

Supplementary Figure 9. a) UPS spectra of ITO coated with a single-layer ZnO and the ZnO stack with 6 layers of ZnO, annealed at 320 ºC, modified by PEIE. **b)** Band diagram for the cathode interfaces in the PBDB-T:ITIC solar cells based on ZnO, PEIE modified ZnO, and ITO/ZnO.

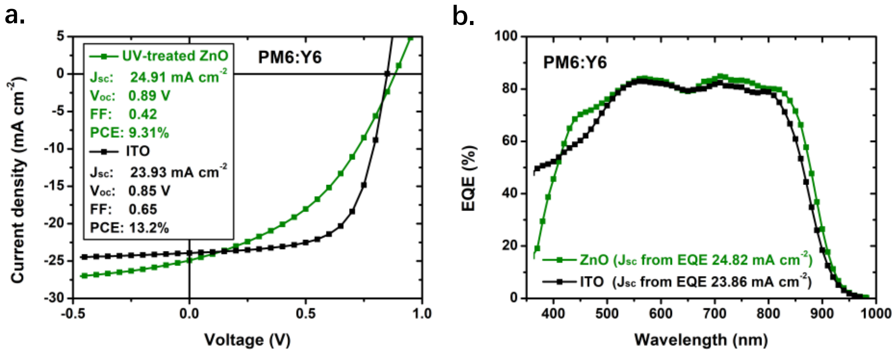

Supplementary Figure 10. a) *J-V* curves and **b)** EQE spectra for the solar cells based on the UV-treated ZnO and non-UV-treated ITO, with the active layers based on PM6:Y6.

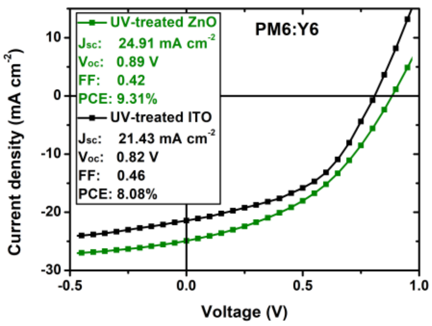

Supplementary Figure 11. *J-V* curves and the performance parameters for the UV-treated ITO based solar cells, compared to the UV-treated ZnO based solar cells with the active layers based on PM6:Y6.

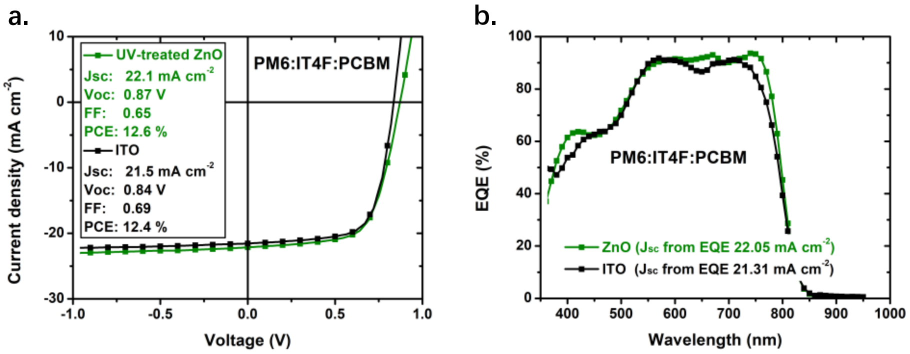

Supplementary Figure 12. a) *J-V* curves and **b)** EQE spectra for the solar cells based on UV-treated ZnO and non-UV-treated ITO, with the active layers based on PM6:IT4F:PCBM.

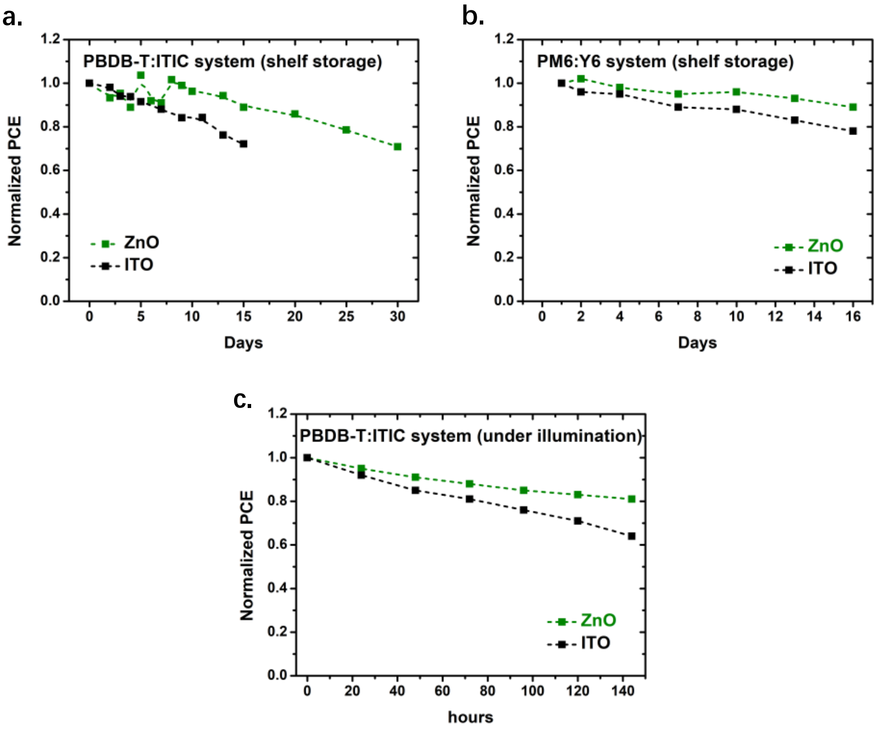

Supplementary Figure 13. Degradation of the UV-treated, encapsulated **a)** PBDB-T:ITIC and **b)** PM6:Y6 solar cells based on the ZnO stack with 6 layers of ZnO, annealed at 320 ºC, compared to that of the ITO based solar cells, stored in air. The solar cells were encapsulated. Only one UV treatment was performed for the ZnO based solar cell after the completion of device construction. The better stability of the ZnO based solar cell indicates that the conductivity of the ZnO stack in the encapsulated device is persistently high, in contrast to that of the air-exposed non-encapsulated ZnO stack, requiring UV reactivation to remain high. **c)** Degradation of the UV-treated encapsulated PBDB-T:ITIC solar cells based on ZnO and ITO under continues solar illumination (100 mW cm^{-2}).

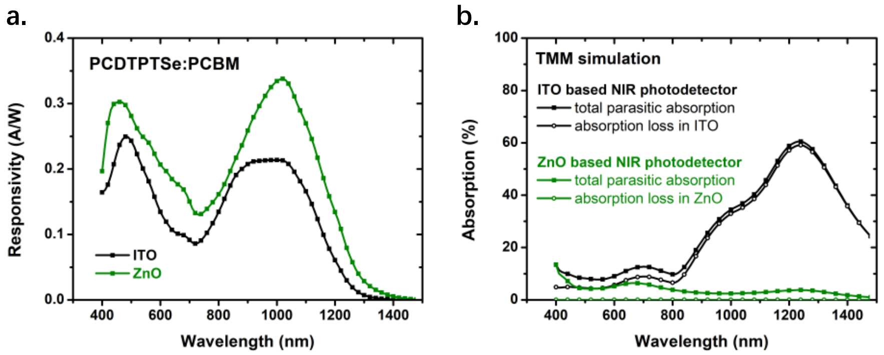

Supplementary Figure 14. **a)** Responsivity of near-infrared (NIR) photodetectors with device architectures of glass/ZnO stack (150 nm)/PEIE/PCDTPTSe:PCBM (80 nm)/MoO_3 (10 nm)/Ag (120 nm) and glass/ITO (170 nm)/single-layer ZnO (30 nm)/PCDTPTSe:PCBM (80 nm)/MoO_3 (10 nm)/Ag (120 nm), measured under a short-circuit condition. The ZnO based device is UV-treated. The increased NIR spectral responsivity of the ZnO based photodetector, compared to that of the ITO based photodetector, is ascribed to the reduced parasitic absorption losses in the electrodes, as confirmed by **b)** the transfer matrix model simulation (TMM) [*J. Appl. Phys.* 86, 487–496 (1999)] results for the NIR photodetectors based on ZnO and ITO, done using the real dielectric constants of the materials.

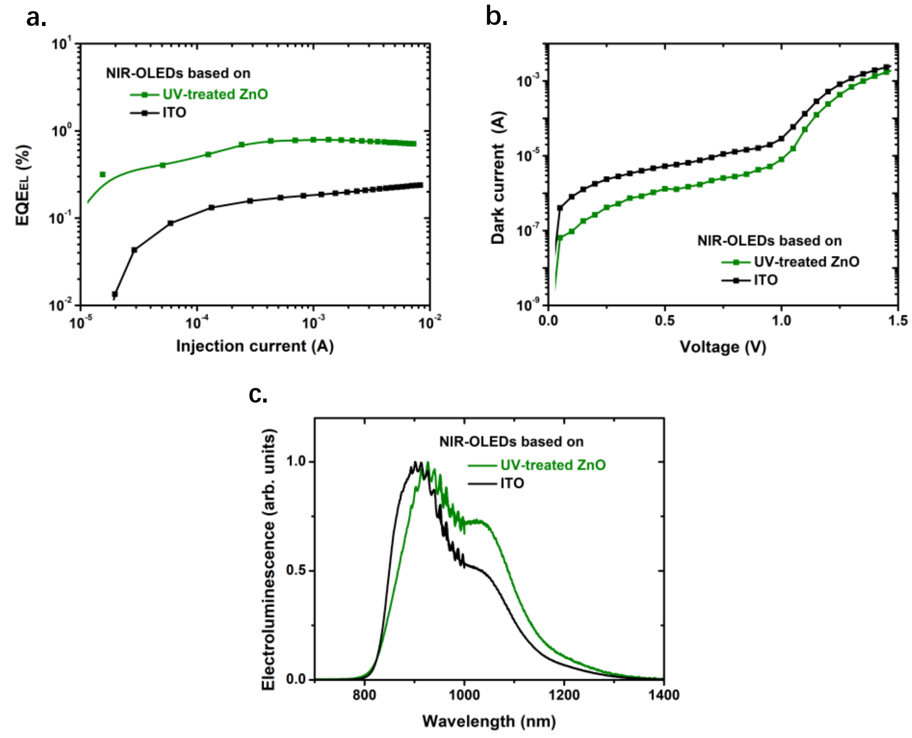

Supplementary Figure 15. **a)** Electroluminescence external quantum efficiency (EQE_{EL}) of near-infrared (NIR) organic light emitting diodes (OLEDs) based on ZnO and ITO, with an emission wavelength over 900 nm. The device architectures are: glass/ZnO stack (150 nm)/PEIE/active layer (Y5, 80 nm)/MoO_3 (10 nm)/Ag (120 nm) and glass/ITO (170 nm)/single-layer ZnO (30 nm)/active layer (Y5, 80 nm)/MoO_3 (10 nm)/Ag (120 nm). The ZnO based device is UV-treated. The increased quantum efficiency of the OLED based on ZnO, compared to that based on ITO, could also be ascribed to the suppressed non-radiative surface recombination loss of charge carriers. **b)** Dark current-voltage characteristic curves of the NIR OLEDs based on ZnO and ITO. **c)** Electroluminescence (EL) spectra of the devices based on ZnO and ITO.

Supplementary Table 1. Statistical photovoltaic performance parameters for the solar cells constructed in this work. The average values and the errors are derived from 10 devices fabricated using the same processing condition.

**Active layer**	**Transparent electrode**	J_{sc} **(mA cm^{-2})**	V_{oc} **(V)**	**FF**	**PCE (%)**
PBDB-T:ITIC	non-UV-treated ITO	15.5±0.5	0.91±0.01	0.64±0.02	9.7±0.5
	UV-treated ZnO	16.7±0.8	1.00±0.02	0.56±0.04	9.7±0.6
PM6:Y6	non-UV-treated ITO	23.1±0.8	0.83±0.02	0.63±0.03	12.7±0.5
	UV-treated ZnO	23.7±1.2	0.89±0.01	0.41±0.04	8.5±0.8
PM6:IT4F:PCBM	non-UV-treated ITO	20.9±0.6	0.83±0.01	0.69±0.01	11.9±0.5
	UV-treated ZnO	21.4±0.7	0.85±0.02	0.62±0.03	12.0±0.6
