# Supplementary material for: A Transparent Electrode Based on Solution-Processed ZnO for Organic Optoelectronic Devices
Source: Nat Commun. 2022 Jul 28;13:4387. doi: 10.1038/s41467-022-32010-y (PMC9334612; doi:10.1038/s41467-022-32010-y)
Supplement: Supplementary file 3 — Solar Cells Reporting Summary [file 41467_2022_32010_MOESM3_ESM.pdf]

nature research

Corresponding author(s): Zaifei Ma, Zheng Tang, Wolfgang Tress

Solar Cells Reporting Summary

Nature Research wishes to improve the reproducibility of the work that we publish. This form is intended for publication with all accepted papers reporting the characterization of photovoltaic devices and provides structure for consistency and transparency in reporting. Some list items might not apply to an individual manuscript, but all fields must be completed for clarity.

For further information on Nature Research policies, including our data availability policy, see Authors & Referees.

Experimental design

Please check: are the following details reported in the manuscript?

1. Dimensions

Item	Yes	No	Details
Area of the tested solar cells	☒	☐	Methods
Method used to determine the device area	☒	☐	Methods

2. Current-voltage characterization

Item	Yes	No	Details
Current density-voltage (J-V) plots in both forward and backward direction	☐	☒	No difference observed for the JV curves measured in forward and backward directions
Voltage scan conditions *For instance: scan direction, speed, dwell times*	☐	☒	No difference observed for the JV curves measured with different scan conditions
Test environment *For instance: characterization temperature, in air or in glove box*	☒	☐	Methods
Protocol for preconditioning of the device before its characterization	☒	☐	Methods
Stability of the J-V characteristic *Verified with time evolution of the maximum power point or with the photocurrent at maximum power point; see ref. 7 for details.*	☒	☐	supplementary information

3. Hysteresis or any other unusual behaviour

Item	Yes	No	Details
Description of the unusual behaviour observed during the characterization	☐	☒	No unusual behavior observed during the measurement
Related experimental data	☐	☒	No unusual behavior observed during the measurement

4. Efficiency

Item	Yes	No	Details
External quantum efficiency (EQE) or incident photons to current efficiency (IPCE)	☒	☐	Figure 6
A comparison between the integrated response under the standard reference spectrum and the response measure under the simulator	☒	☐	Figure 6
For tandem solar cells, the bias illumination and bias voltage used for each subcell	☐	☒	No tandem solar cells studied in this work

5. Calibration

Item	Yes	No	Details
Light source and reference cell or sensor used for the characterization	☒	☐	Methods
Confirmation that the reference cell was calibrated and certified	☒	☐	Methods

Calculation of spectral mismatch between the reference cell and the devices under test

☒ Yes ☐ No — Methods

6. Mask/aperture

Size of the mask/aperture used during testing

☒ Yes ☐ No — Methods

Variation of the measured short-circuit current density with the mask/aperture area

☐ Yes ☒ No — The reported short-circuit current density values were compared to the current density derived from the sensitive EQE measurements (measured with a bias illumination intensity of 100 mW cm-2).

7. Performance certification

Identity of the independent certification laboratory that confirmed the photovoltaic performance

☐ Yes ☒ No — This work does not report high power conversion efficiency values, but focuses on the device operation mechanism.

A copy of any certificate(s)
Provide in Supplementary Information

☐ Yes ☒ No — This work does not report high power conversion efficiency values, but focuses on the device operation mechanism.

8. Statistics

Number of solar cells tested

☒ Yes ☐ No — supplementary information

Statistical analysis of the device performance

☒ Yes ☐ No — supplementary information

9. Long-term stability analysis

Type of analysis, bias conditions and environmental conditions
For instance: illumination type, temperature, atmosphere humidity, encapsulation method, preconditioning temperature

☒ Yes ☐ No — supplementary information
